# Treatment of an endodontic-periodontal lesion using peripheral blood mesenchymal stem cells (PBMSCs) and platelet-rich fibrin matrix (PRFM): A case report

**DOI:** 10.34172/japid.025.3502

**Published:** 2025-03-08

**Authors:** Sphoorthi Anup Belludi, Sharaz Shaik, Neha Pradhan, Sreeparvathy Rema

**Affiliations:** ^1^Department of Periodontics, K.L.E Society’s Institute of Dental Sciences, Bengaluru, Karnataka, India; ^2^Lincoln University PhD Program, Lenora Institute of Dental Science, Rajahmundry, Andhra Pradesh, India

**Keywords:** Endo-periodontal lesion, Peripheral blood stem cells, Platelet-rich fibrin, Regenerative endodontics

## Abstract

In the current report, we discuss the available treatment options and present a successfully treated periodontal-endodontic lesion using autogenous peripheral blood mesenchymal stem cells (PBMSCs) and platelet-rich fibrin matrix (PRFM). A patient presented with a complaint of food impaction and bad breath. Clinically, the lower right first molar was non-vital and had a deep periodontal pocket and attachment loss. Radiographically, the distal root had an angular bone loss extending to the apex. The endodontic condition was treated with chemomechanical debridement, calcium hydroxide dressing, and obturation. Later, we reflected a full-thickness mucoperiosteal flap and thoroughly debrided the granulation tissue. We filled the defect with a gel containing PBMSCs and PRFM, prepared from the patient’s peripheral blood, and sutured the flap. After nine months, we noticed significant osseous fill and 5 mm of gain in the clinical attachment level. The outcomes of the case show the periodontal regenerative potential of the novel combination.

## Introduction

 The concomitant presence of inflammatory periodontal disease and pulpal pathosis complicates the diagnosis and treatment planning of endodontic-periodontal lesions (EPLs). Hence, EPLs usually need a multidisciplinary approach.^1,2^ The aim of treating the periodontal pocket is to regain the periodontal attachment; it may range from oral prophylaxis to regenerative procedures. Currently, much interest has been observed in autologous platelet/fibrin biologics. According to Dohan’s classification, our material of interest is pure platelet-rich fibrin/platelet-rich fibrin matrix (PRFM).^3,4^

 In alignment with regenerative dentistry and the current concept of tissue engineering, stem cells can be an effective module due to their pluripotency and regenerative ability.^5^ The extraction of MSCs from bone marrow and other sources is an invasive, high-risk approach that provides a low-frequency and heterogeneous population. Peripheral blood is possibly the most straightforward source due to the accessibility and lack of donor-organ morbidity in comparison to other sources (bone marrow, umbilical cord, adipose tissue, salivary glands, periosteum, periodontium, and dental pulp) of mesenchymal stem cells (MSCs). Despite numerous studies on using other sources of stem cells in periodontal regeneration,^5^ there is a paucity of reports on the application of peripheral blood-derived mesenchymal stem cells (PBMSCs). The current case report describes a novel approach using PBMSCs + PRFM en masse to treat an EPL patient.

## Case Presentation

**Figure 1:**
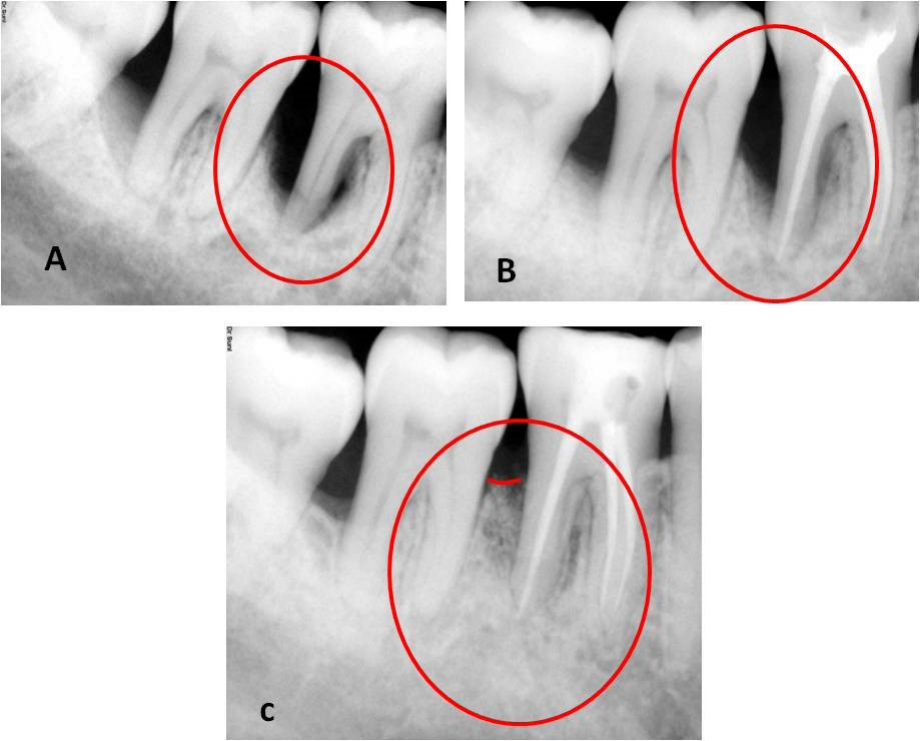
Preoperative, postoperative, and follow-up radiographs. (A) Preoperative radiograph displaying endodontic-periodontal lesion. (B) Three-month follow-up displaying partial bone regain. (C) Nine-month follow-up displaying a significant bone regain

 A healthy patient (male, age = 42 years) presented with a chief complaint of discomfort and food impaction in the lower right back teeth region and bad breath for five months. Intraoral examination revealed a full constituent dentition, generalized moderate accumulation of dental biofilm, calculus, and generalized bleeding on probing, more prominent in the lower right second premolar to the third molar region (#45 to #48). Food impaction was observed between the lower right first and second molars (#46 and #47). Mild tenderness on percussion (TOP) was elicited with #46 and #47. Grade I mobility and occlusal and buccal caries were detected with #46. The clinical attachment level (CAL) and probing pocket depth (PPD) were 8 mm in the interdental region of #47 and #48 and 4 mm between #45 and #46. However, the CAL was 9 mm, and PPD was 8 mm in the interdental region of #46 and #47 due to 1 mm of gingival recession on the distobuccal root of #46 (Table 1). A long-cone paralleling technique and intraoral direct digital periapical radiovisiograph (RVG-Suni Medical Imaging, Apteryx Inc., Acron, Ohio, USA) were used for radiographic evaluation (Table 2). The radiographs revealed angular bone loss in the lower right first and second molar (#46 and #47) region, with the base of the vertical defect extending to the apex of the distal root of the lower right first molar (#46); however, there was no sign of root damage (Figure 1). Vitality test exhibited a delayed response with #46 and a positive response with #47. The patient was devoid of complicating factors such as diabetes and smoking, which could have affected the healing and treatment outcome. Based on the presentations and common classification by Simon et al.,^6^ the lesion was classified as primary periodontal lesions with secondary endodontic involvement and grade 3 endo-periodontal lesion in periodontitis patients as per the 2017 World Workshop Classification.^7^

## Treatment

 Regarding the clinical and radiographic findings, the provisional prognosis was considered fair. The treatment protocol was planned in 4 phases^8^: (1) preliminary management, (2) endodontic management, 3: PBMSCs + PRFM gel preparation and surgical procedure, and (4) follow-up management.

###  Preliminary management and patient consent 

 We performed oral prophylaxis, educated the patient about the importance of oral hygiene, and demonstrated the use of dental floss and chlorhexidine mouthwash. Since the patient did not have acute symptoms, we did not prescribe oral medications. We explained the current condition of the lower right first molar (#46) and our proposal to use the novel treatment procedure. The patient voluntarily gave consent for the procedure. Written informed consent was obtained from the patient for enrollment into the treatment protocol and for publishing the acquired data. Ethical clearance for the treatment was received from the Institutional Ethics Committee (Blinded for review).

###  Endodontic management 

 Root canal treatment incorporated chemomechanical debridement under local anesthesia and isolation using rotary endodontic instruments (ProTaper Next, Dentsply Sirona) and filling of the root canals with calcium hydroxide paste (ApexCal® Ivoclar Vivadent) using a lentulo spiral. The root canals were obturated by lateral condensation of gutta-percha (ProTaper Next Gutta Percha endodontic points, Dentsply, Maillefer) and a calcium hydroxide-based sealer (Apexit Plus Root Canal Sealer, Ivoclar Vivadent Inc.). A permanent composite resin restoration was placed to seal off the access cavity.

###  PBMSCs + PRFM gel preparation^9^

**Figure 2:**
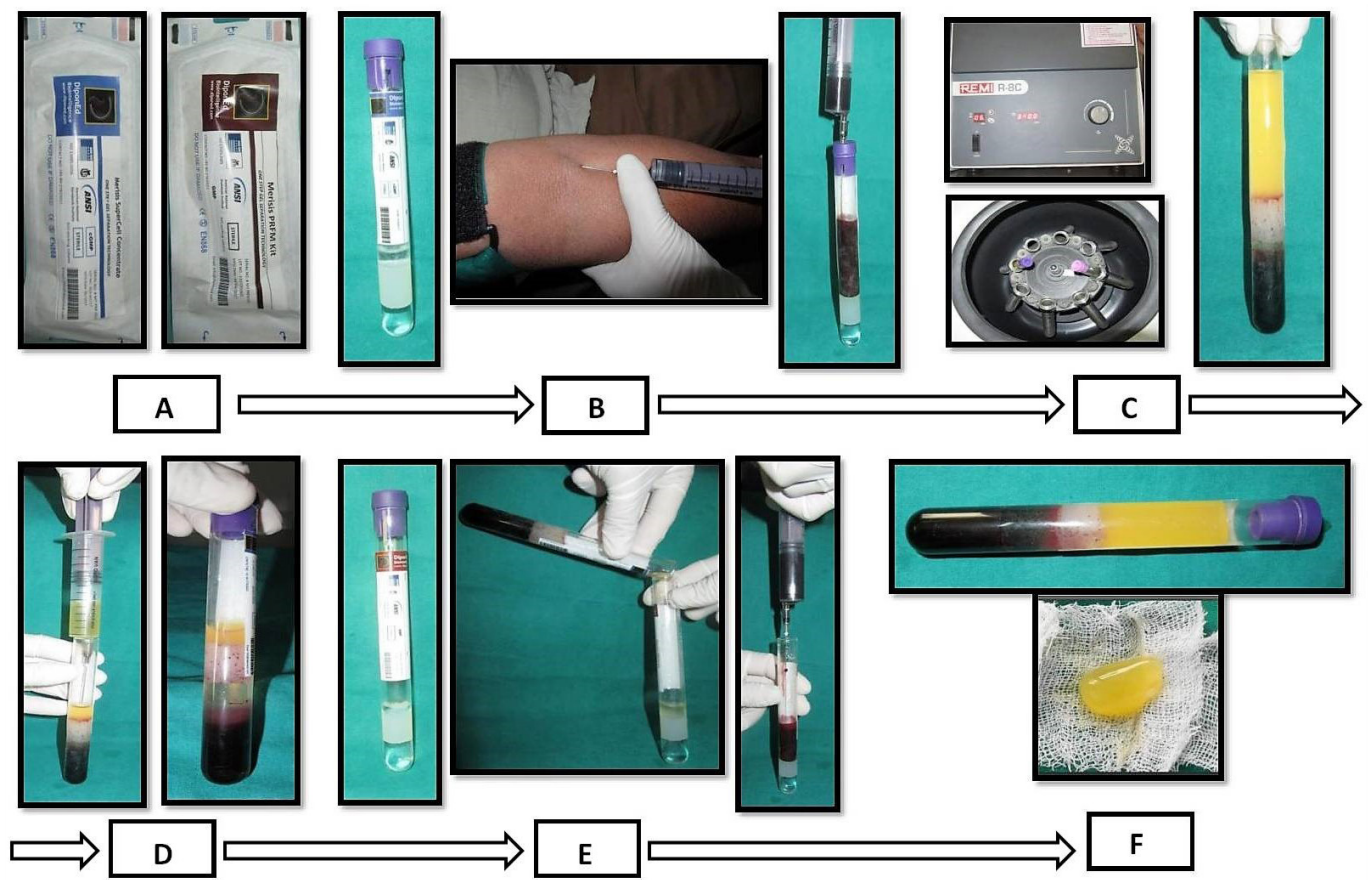
Procurement and preparation of PBMSCs + PRFM en masse. (A) Merisis kit for PRFM and Merisis Supercell concentrate kit for PBMSCs. (B) Aspiration of blood for stem cell preparation. (C) Centrifugation of blood in a laboratory centrifuge. (D) Extraction of PBMSCs from the uppermost layer of 0.5-1 mL of fluid from the kit. (E) The second aspiration of blood for PRFM preparation. (F) PRFM and PBMSCs en masse obtained

 PBMSCs and PRFM were obtained from the peripheral blood by Merisis Supercell concentrate, DiponEd BioIntelligence© and Merisis PRFM kit, Merisis Biological Devices, and DiponEd BioIntelligence©, respectively, by following manufacturer kit insert instructions (Figure 2).^9^

###  Surgical procedure

**Figure 3:**
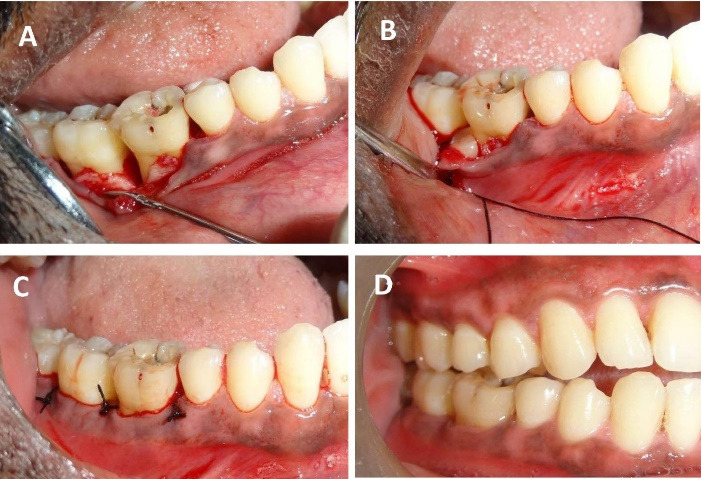
Surgical procedure and post-surgical illustrations. (A) A mucoperiosteal flap was reflected, and surgical debridement was performed. (B) PBMSCs + PRFM en masse was placed in the intrabony defect. (C) Interrupted sutures were placed to approximate the flap. (D) Three-month postoperative follow-up displaying complete soft tissue healing

 After anesthetizing with 2% lignocaine containing 1:80,000 adrenaline (Lignox A 2%, Indoco Remedies LTD., India) using the inferior alveolar nerve block. We placed sulcular incisions on both buccal and lingual sides and reflected a full-thickness mucoperiosteal flap extending from the mesial of the lower right second premolar (#45) to the mesial line angle of the lower right third molar (#48). Thorough debridement of granulation tissue in the intrabony defect (3-wall defect between #46 and #47 and #47 and #48 regions), scaling, and root planing of remnant calculus on the root surfaces was performed (Figure 3). After pre-suturing of the interdental papillae in the #46, #47, and later in #47 and #48 regions, the freshly prepared PBMSCs + PRFM was extracted en masse from the glass vial with the help of tweezers and filled into the intrabony defect as a sole material. The flaps were approximated by interrupted sutures using 4-0 silk material (Figure 3). No periodontal dressing was applied. Antibiotics (Amoxycillin 500 mg every 8 hours for 7 days) and analgesics (Ibuprofen 400 mg every 4 hours) as required for pain management were prescribed. 0.2% chlorhexidine rinses every 12 hours for 14 days were advised. Postoperative instructions were given, an ultra-soft toothbrush was prescribed, and the patient was advised to refrain from disturbing the surgical site for the next two days.

###  Outcome and follow-up

 The patient was followed for one month after endodontic treatment with no evident changes in the clinical parameters; hence, we proceeded to surgical therapy. We also conducted post-surgical follow-up after a week, one month, 3 months, and 9 months. During the first follow-up appointment, the patient had no complaints of pain and discomfort and no tenderness on percussion. We reinforced oral prophylactic instructions. The sutures were removed ten days after surgery. During the one-month and three-month appointments, the patient displayed good oral hygiene. The surgical area displayed complete healing, no tenderness on percussion, and no tooth mobility. Radiographic evaluation three months after surgery displayed a partial bone fill (Figure 1). During the 9-month follow-up appointment, the periodontal lesion had significantly improved clinically and radiographically (Figures 1 and 3). A significant reduction in the periodontal probing depth and gain in the clinical attachment level was noted (Table 1). At any of these intervals, there was no attempt at subgingival instrumentation.

**Table 1:** Clinical attachment loss at right lower quadrant, measured in mm: a pre- and 9-month postoperative comparison

	**Tooth no.**															
	**#45**				**#46**				**#47**				**#48**			
	**Pre**		**Post**		**Pre**		**Post**		**Pre**		**Post**		**Pre**		**Post**	
Buccal	3		3		3		3		3		3		3		3	
Lingual	3		3		4		3		3		3		3		3	
Interdental	M	D	M	D	M	D	M	D	M	D	M	D	M	D	M	D
	3	4	2	3	4	9	3	4	8	8	4	4	7	3	3	3

Pre: preoperative parameters (in mm). Post: 9-month postoperative parameters (in mm). M: mesial, D: distal.

**Table 2:** Standardized parameters used for pre- and postoperative radiographic evaluation

**Parameter**	**Description**
Type of radiograph	Intraoral direct digital periapical radiovisiograph
Radiographic equipment	RVG-Suni Medical Imaging, Apteryx Inc., Acron, Ohio, USA.
Technique	long cone paralleling technique
Exposure	70 kVp, 7 ma for 0.2 seconds
The focus-to-film distance	20 cm
Software for linear measurements	Image J software, Wayne Rasband, National Institute of Health, USA

## Discussion

 A combined EPL does not respond with root canal treatment alone. The patient’s 4-week post-root canal treatment follow-up evaluation did not indicate any distinct improvement in the clinical parameters. Considering CAL deeper than the critical probing depth of 5.4 mm in our case, surgical intervention with regenerative therapy was deemed beneficial.^10^ In the current case, after initial biomechanical debridement, the canals were filled with calcium hydroxide endodontic dressing for a week, followed by obturation with gutta-percha and a calcium hydroxide-based sealer. A report suggests that pulpal infection tends to stimulate epithelial growth apically adjacent to the stripped dentinal surface, especially in the presence of periodontal infection.^10,11^ Open dentinal tubules, accessory canals, and apical foramen are the three possible transmission channels for contamination.^12^

 In the present case, as the vitality test showed a delayed response, endodontic therapy was carried out to eliminate the partially necrotic pulp and prevent remnant nidus of infection and future necrosis and infection following periodontal regenerative surgery. Therefore, root canal treatment was completed before periodontal regenerative therapy. Furthermore, calcium hydroxide dressing in the root canal could promote periodontal healing in such cases.^10^ The overall success of an EPL usually depends on the efficiency of periodontal therapy.

 Numerous biomaterials are currently available for periodontal regeneration. A study reported that a combination of PRP and stem cells resulted in better periodontal tissue regeneration than stem cell therapy alone^13^; furthermore, using PRFM instead of PRP could produce better results.^14,15^ PRFM enhances osteoblast differentiation and stimulates periodontal soft tissue regeneration.^16^ The influence of PRFM on periodontal regeneration can be accredited to transforming growth factor β, platelet-derived growth factor, insulin-like growth factor, and basic fibroblast growth factor.^15,17^ Apart from the healing potential of PRFM, it was shown that PRFM produced significantly higher mesenchymal stem cell proliferation.^17^ There are studies on the application of stem cells from various sources, more so on bone marrow mesenchymal stem cells;^5^ however, little is known on tissue regeneration by PBMSCs. PBMSCs have equivalent pluripotency and osteogenic ability to mesenchymal stem cells derived from bone marrow and umbilical cord.^18,19^ The stem cell-based periodontal regeneration could be mediated by monocyte chemoattractant protein-1, chemokine stromal cell-derived factor-1, chemokine stromal cell-derived factor-1, and exosomes cell-free strategy.^20^ Combining PRFM with PBMSCs would be more salutary than PRP as there is a more sustained release of growth factors by PRFM in comparison to PRP.^21,22^ Our approach of using PBMSCs + PRFM en masse in periodontal regeneration of EPL is novel as there are no similar reports in the current scientific literature. However, few studies suggest promising results for this combination as a regenerative material.^9,13^

 The success rate of conventional modalities in the treatment of EPL combined lesions was reported to be very low (27‒37%) compared to a tooth survival rate of 92.31% at 5 years after a periodontal regenerative procedure.^23,24^Studies suggest that treatment outcomes seen at a first-year follow-up appointment tend to last for a long time.^25^ Our 9-month follow-up revealed an excellent treatment outcome. Uniformity in pre- and postoperative radiography and analysis were used to minimize the errors (Table 2). No bone graft or radiopaque material was used, which might mimic radiopaque bone fill in postoperative radiographs. Hence, the postoperative radiopacity can be considered a true osseous fill.

## Conclusion

 A substantial osseous fill in the present study can be attributed to a definitive root canal treatment, periodontal debridement, and application of PRFM + PBMSCs. This protocol can be an easy, effective, and promising regenerative option. However, in vitro validity tests, histomorphometry studies, and large-scale randomized control trials are required to endorse the regenerative efficacy of this protocol.

## Competing Interests

 The authors do not have any financial interest in the companies whose materials are included in this article and have no conflicts of interest.

## Consent for Publication

 Not applicable.

## Data Availability Statement

 Not applicable.

## Ethical Approval

 We explained the current condition and our proposal for using the novel treatment procedure. The patient voluntarily gave consent for the procedure. Written informed consent was obtained from the patient for enrollment into the treatment protocol and for publishing the acquired data. Ethical clearance for the treatment was received from the Institutional Ethics Committee (IEC/Nov-2021/8).
